# How Close Are We to Achieving Durable and Efficacious Gene Therapy for Hemophilia A and B?

**DOI:** 10.3390/genes16101200

**Published:** 2025-10-14

**Authors:** Patrycja Sosnowska-Sienkiewicz, Danuta Januszkiewicz-Lewandowska

**Affiliations:** 1 Department of Pediatric Surgery, Medical University of Warsaw, Żwirki i Wigury 63A Street, 02-091 Warsaw, Poland; 2 Department of Pediatric Oncology, Hematology and Transplantology, Poznan University of Medical Sciences, ul. Fredry 10, 61-701 Poznan, Poland; danuta.januszkiewicz@ump.edu.pl

**Keywords:** hemophilia A, hemophilia B, factor VIII deficiency, factor IX deficiency, gene therapy

## Abstract

Hemophilia, an X-linked recessive bleeding disorder, results from mutations in the *F8* or *F9* genes, leading to factor VIII (hemophilia A) or factor IX (hemophilia B) deficiency. While conventional treatment relies on regular factor replacement therapy, gene therapy has emerged as a promising alternative, offering the potential for sustained endogenous factor production after a single administration. This review provides an in-depth analysis of recent advances in gene therapy for both hemophilia A and B, with a focus on AAV-mediated liver-directed approaches and other approved modalities. Key limitations—such as vector immunogenicity, hepatic toxicity, waning transgene expression, and limited re-dosing capacity—are discussed. Additional gene delivery platforms, including lentiviral and retroviral vectors, genome editing techniques (e.g., CRISPR/Cas9), and non-viral systems like transposons and lipid nanoparticles, are also examined. Although gene therapy for hemophilia B demonstrates greater clinical durability, hemophilia A presents unique challenges due to factor VIII’s size, poor expression efficiency, and the need for higher vector doses. Future efforts will focus on overcoming immune barriers, improving delivery technologies, and developing approaches suitable for pediatric patients and individuals with pre-existing immunity. This review provides not only a descriptive overview but also a critical comparison of gene therapy approaches for hemophilia A and B. We emphasize that the durability of response is currently superior in hemophilia B, whereas hemophilia A still faces unique barriers, including declining FVIII expression and higher immunogenicity. By analyzing cross-platform challenges (AAV, lentiviral, CRISPR, and emerging LNPs), we highlight the most promising strategies for overcoming these limitations and provide a forward-looking perspective on the future of gene therapy.

## 1. Introduction

Hemophilia refers to a rare genetic coagulopathy characterized by impaired hemostatic response due to insufficient levels or functional defects of clotting proteins. The condition is primarily categorized into two clinical entities: hemophilia A, associated with mutations in the *F8* gene encoding coagulation factor VIII, and hemophilia B, resulting from pathogenic variants in the *F9* gene responsible for factor IX synthesis. Both factors are essential cofactors in the intrinsic pathway of the coagulation cascade, facilitating the efficient generation of thrombin and the formation of a stable fibrin clot [1,2]. These mutations are typically inherited in an X-linked recessive pattern, meaning the disease predominantly affects males, while females are most often asymptomatic carriers. However, due to lyonization (random X-chromosome inactivation), some females with hemophilia may exhibit bleeding [3].

The defective or insufficient clotting factors in hemophilia disrupt the normal formation of thrombin and stable fibrin clots, leading to prolonged or spontaneous bleeding episodes. Clinically, this may manifest as easy bruising, prolonged bleeding after minor injuries or surgeries, hematuria, hematomas, or spontaneous hemorrhages—particularly into joints (hemarthroses) and muscles. Recurrent joint bleeds, if untreated, can result in chronic synovitis, progressive arthropathy, muscle contractures, and long-term disability [4].

The severity of the disease correlates with the residual factor activity in plasma. In severe hemophilia (<1% of normal factor activity), patients may experience spontaneous bleeding early in life, chronic joint damage, muscle contractures, and complications from repeated infusions [5,6]. Milder deficiencies (factor activity 5–40%) typically manifest as post-traumatic or post-operative bleeding in later stages of life [7].

The diagnostic workup for hemophilia involves a comprehensive clinical assessment alongside targeted laboratory investigations. Initial coagulation studies typically reveal a prolonged activated partial thromboplastin time (aPTT) with a normal prothrombin time (PT), suggesting intrinsic pathway dysfunction. Confirmation requires quantitative measurement of factor VIII or IX activity [4]. Genetic testing plays a crucial role in establishing a definitive diagnosis, facilitating carrier detection in female relatives, predicting inhibitor risk, and enabling prenatal counseling. Furthermore, molecular diagnostics allow clinicians to differentiate hemophilia from phenotypically similar bleeding disorders, such as von Willebrand disease type 2N, and guide personalized treatment strategies [4,8].

Traditional management of hemophilia has relied on replacement therapy, involving intravenous infusions of recombinant or plasma-derived clotting factor concentrates. While effective in reducing bleeding episodes, this approach requires frequent administration, poses risks of inhibitor formation, and is often associated with high treatment costs and logistical challenges, especially in low-resource settings [8,9]. Table 1 presents a comparison of the benefits and risks associated with different gene therapy strategies for hemophilia (Table 1).

If left untreated or inadequately managed, hemophilia can lead to life-threatening hemorrhages, irreversible joint damage, intracranial bleeding, and complications related to repeated exposure to blood products. Advances in recombinant factor concentrates, prophylactic regimens, and gene therapy have significantly improved life expectancy and quality of life for patients, yet access to optimal care remains variable worldwide [7,8].

The aim of this article is to provide a comprehensive overview of current therapeutic approaches to gene therapy in hemophilia A and B, with particular emphasis on their clinical efficacy and the challenges associated with implementation.

## 2. Gene Therapy

In vivo gene transfer using adeno-associated virus (AAV) vectors to hepatocytes has emerged as the most promising approach, forming the basis of numerous advanced clinical trials and leading to the regulatory approval of two products for the treatment of hemophilia B and one for the treatment of hemophilia A [10].

## 3. Hemophilia A

Hemophilia A is an X-linked recessive bleeding disorder caused by mutations in the *F8* gene and affects approximately 1 in 5000 live male births worldwide [11].

### 3.1. AAV Vector and Its Properties

Adeno-associated virus (AAV) is a non-pathogenic, single-stranded DNA virus that, after modification (rAAV), is used as a gene delivery vehicle in gene therapy. Its advantages include low immunogenicity, the ability to infect both dividing and non-dividing cells, and the potential to persist in cells as an episome, thereby reducing the risk of insertional mutagenesis. A major limitation, however, is its relatively small packaging capacity of approximately 5 kilobases (kb) [12].

### 3.2. Mechanism and Therapeutic Approach

Gene therapy for hemophilia A primarily employs adeno-associated viral (AAV) vectors to deliver the F8 transgene—most commonly in the B-domain-deleted (BDD-FVIII) form—directly to hepatocytes. Upon entry into the cell nucleus, the transgene is transcribed, leading to endogenous production of coagulation factor VIII [13].

### 3.3. Approved Therapies and Their Effectiveness

The first gene therapy approved for adult patients with hemophilia A is Roctavian (valoctocogene roxaparvovec), based on an AAV5 vector encoding an optimized BDD-FVIII gene. A single intravenous infusion significantly reduces the annualized bleeding rate (ABR) and enables most patients to discontinue prophylactic factor VIII replacement therapy. However, circulating FVIII levels often decline over time, with some individuals falling below 5% of normal activity within 4–5 years after treatment [10].

### 3.4. Roctavian (Valoctocogene Roxaparvovec)

Roctavian utilizes a recombinant AAV5 vector that delivers an optimized B-domain-deleted factor VIII (BDD-FVIII) transgene to hepatocytes. Once the genetic material is delivered into the nucleus of the hepatocyte, transcription and translation occur, leading to endogenous production of factor VIII in the liver [14].

In the pivotal GENEr8-1 study that supported regulatory approval, a single infusion of Roctavian significantly reduced the annualized bleeding rate (ABR) and enabled most patients to discontinue prophylactic factor VIII replacement. Many participants achieved FVIII activity levels above 5%, with some reaching levels consistent with mild hemophilia or even within the normal range [10,15].

The most common adverse event was a transient elevation of liver enzymes (ALT/AST), observed in over 80% of patients, which was managed with glucocorticoid therapy. A gradual decline in FVIII expression over time remains a major limitation—after 2–4 years, many individuals had FVIII activity levels below 5%, potentially impacting the long-term durability of the therapeutic effect. Additionally, re-administration of the therapy is currently not possible due to the development of neutralizing antibodies against AAV5, which limits the potential for repeat dosing to boost therapeutic levels. Currently, AAV-based gene therapies are not recommended for pediatric patients due to a theoretical loss of efficacy in the developing liver [16].

Roctavian is approved for use in adult patients with severe hemophilia A who do not have active FVIII inhibitors or significant liver pathology and who lack high titers of anti-AAV5 antibodies. This therapy marks a new era in the management of hemophilia, offering the potential for long-term benefit with a single administration, though its sustained efficacy over time remains an active area of investigation [14,16].

The GENEr8-1 phase 3 trial enrolled 134 adult males with severe hemophilia A (FVIII activity < 1%) who were previously receiving prophylactic FVIII therapy. Participants received a single intravenous dose of 6 × 10^13^ vg/kg of valoctocogene roxaparvovec. The efficacy outcomes are as follows: at week 104, the median FVIII activity level (measured by chromogenic assay) was approximately 7.5 IU/dL, indicating sustained endogenous FVIII production. The bleeding outcomes are as follows: the mean ABR decreased from 5.4 (baseline) to 0.7 bleeds per year. The prophylaxis discontinuation is as follows: over 90% of participants discontinued routine FVIII prophylaxis after gene therapy administration. The durability is as follows: although FVIII levels gradually declined over time, the majority of participants maintained a clinically meaningful reduction in bleeding events during the follow-up period [15].

### 3.5. Challenges and Limitations

The reduction in FVIII expression may be attributed to immune responses, endoplasmic reticulum stress, or epigenetic silencing. Recent studies have provided more detailed insight into the mechanisms underlying the decline in FVIII expression. Epigenetic silencing, including DNA methylation and histone modifications, has been implicated in reducing transgene transcription over time. In addition, endoplasmic reticulum (ER) stress caused by FVIII misfolding can impair hepatocellular protein processing and diminish the secretion of functional FVIII. Finally, immune-mediated clearance of transduced hepatocytes by cytotoxic T cells may contribute to progressive loss of expression. To address these problems, new strategies are under investigation, such as capsid variants with reduced immunogenicity, bioengineered FVIII constructs optimized for folding and secretion, and alternative delivery methods, including lipid nanoparticles (LNPs) and ex vivo modified hepatocytes. These approaches may help overcome the durability gap between hemophilia A and hemophilia B gene therapies [14,16,17]. Most patients experience transient elevations in liver transaminases, and corticosteroid therapy is commonly administered to mitigate potential immune-mediated hepatotoxicity. Another major limitation is the considerable inter-individual variability in therapeutic response, as well as the inability to re-administer the AAV vector due to the development of long-lasting neutralizing antibodies [14,16,17].

### 3.6. Future Directions and Improvements

Ongoing research is focused on developing FVIII variants with enhanced stability and secretion efficiency, such as those incorporating modified porcine sequences (e.g., X5) or variants resistant to inactivation by activated protein C. Innovative approaches also include gene editing technologies like CRISPR-Cas9 and alternative gene delivery platforms, including lipid nanoparticles (LNPs) and ex vivo gene therapy strategies. Studies have emerged evaluating the suitability of human placental cells (PLCs) as delivery vehicles for FVIII, as well as identifying the optimal FVIII transgene that enables the production and secretion of therapeutic levels of this factor from these cells [14,16,17,18].

### 3.7. Hepatocellular Carcinoma (HCC)

The potential risk of hepatocellular carcinoma (HCC) associated with AAV-based gene therapy has been a concern, but clinical evidence to date does not confirm a causal link in humans. A few isolated cases of HCC have been reported in patients receiving AAV gene therapy, but these individuals often had pre-existing risk factors: chronic hepatitis B or C, alcohol-related liver disease, advanced age, and liver cirrhosis [19].

### 3.8. Lentiviral Vectors

Autologous hematopoietic stem cells (HSCs) were genetically modified using the lentiviral vector CD68-ET3-LV, which encodes a novel F8 transgene (ET3) driven by the myeloid-specific CD68 promoter. Two approaches were applied: without a transduction enhancer (group 1) and with a transduction enhancer (group 2). Following myeloablative conditioning, the transduced HSCs were transplanted into recipients. The therapy was evaluated for both safety—based on engraftment efficiency and conditioning-related toxicity—and efficacy, as measured by factor VIII activity and the annualized bleeding rate. Gene therapy for hemophilia A using lentiviral vector-transduced autologous HSCs resulted in sustained factor VIII expression [2].

### 3.9. Other Gene Therapies

Giroctocogene Fitelparvovec (SB-525) is an experimental gene therapy based on AAV6 vector technology, which delivers an optimized BDD-FVIII gene (B-domain deleted factor VIII) directly to hepatocytes. ASC618 is a second-generation AAV2/8 vector and encodes a liver-specific codon optimized (LCO) bioengineered B-domain deleted hFVIII (ET3) under a synthetic Hepatic Combinatorial Bundle (HCB) promoter (HCB-ET3-LCO) [20]. The GO-8 trial, which used the AAV8-HLP-hFVIII-V3 vector, achieved stable factor VIII expression in most participants with severe hemophilia A over a follow-up period of up to five years [21].

### 3.10. CRISPR-Based Approaches

CRISPR allows targeted genome editing of FVIII/FIX loci. In 2024, a high-activity variant, p18T-BDD-F8-V3, was inserted into the mROSA26 locus in mice using both HDR and NHEJ, achieving stable FVIII expression [22]. The risks associated with CRISPR-Cas9 gene editing include off-target effects and the potential for genotoxicity due to prolonged Cas9 expression. Off-target effects refer to unintended edits at sites in the genome other than the intended target, potentially leading to unforeseen consequences. Prolonged Cas9 expression can also cause genotoxicity, meaning it can damage DNA and potentially lead to mutations or other genomic instability [23,24].

### 3.11. Lipid Nanoparticles (LNPs)

Lipid nanoparticles (LNPs) are a type of non-viral delivery system that offer several advantages, including avoiding size constraints and potentially enhanced safety compared to viral vectors. While LNPs can effectively deliver therapeutic factors like FVIII and demonstrate a relatively safe hepatic profile in mice (no increase in ALT levels), transgene expression can be limited due to epigenetic silencing and cell turnover. LNPs represent a promising avenue for gene therapy and other therapeutic applications, offering advantages in terms of delivery, safety, and versatility. However, ongoing research is crucial to address limitations like transgene expression duration and potential toxicity to optimize their clinical use [25,26].

## 4. Hemophilia B

Hemophilia B, a rare bleeding disorder inherited in an X-linked recessive pattern, arises due to a partial or complete deficiency of coagulation factor IX (FIX). Factor IX plays a crucial role in the coagulation cascade that halts bleeding following injury. Upon vascular injury, factor IX is activated by factor VIIa, which in turn activates factor X. Activated factor X (Xa) converts prothrombin into thrombin, which subsequently transforms fibrinogen into fibrin, forming a stable blood clot. A deficiency in functional factor IX impairs this cascade, resulting in uncontrolled bleeding [12,27,28].

Hemophilia B, which accounts for approximately 15–20% of all hemophilia cases, is significantly less prevalent than hemophilia A, with an estimated incidence of one in 25,000 to 30,000 live male births [11,29].

### 4.1. Gene Therapy

Gene therapy for hemophilia B involves delivering a functional copy of the *F9* gene into hepatocytes, the liver cells responsible for producing factor IX [12]. Of the two hemophilia types, gene therapy for hemophilia B has demonstrated greater efficacy and has progressed further in recent clinical trials. This is largely attributed to the simpler molecular structure of factor IX compared to factor VIII. Moreover, the post-translational processing of factor IX can be efficiently accomplished in skeletal muscle, which enabled early preclinical studies to be conducted in a less critical target tissue than the liver—the natural site of factor IX production- thereby reducing associated risks [27,30].

### 4.2. Early Stages of Gene Therapy for Hemophilia B

Initial attempts focused on delivering the factor IX gene to skeletal muscle. While expression of hFIX was achieved, therapeutic levels were not reached, and immune responses against hFIX posed a significant barrier [31].

### 4.3. Transition to Liver-Directed Gene Transfer

The liver proved to be a more effective target, as it is the natural site of factor IX synthesis. Gene transfer to hepatocytes can induce immune tolerance, primarily mediated by regulatory T cells (Tregs), which limits the development of antibodies and cytotoxic T lymphocyte responses [32].

### 4.4. First Clinical Trials Using AAV2

In clinical trials with AAV2, it was observed that even low levels of neutralizing antibodies could prevent successful transduction. In some patients, a CD8+ T cell response against the capsid emerged, leading to liver damage and loss of factor IX expression [10].

### 4.5. Improvements: AAV8 and Self-Complementary AAV (scAAV) Vectors

AAV8, derived from rhesus macaques, exhibits higher liver tropism and a lower prevalence of neutralizing antibodies in the human population. scAAV vectors contain double-stranded DNA, allowing faster and stronger transgene expression. However, they may also trigger stronger immune responses [33].

### 4.6. Approved Therapies and Their Effectiveness

#### 4.6.1. Hemgenix (Etranacogene Dezaparvovec)

A single intravenous infusion of an rAAV5 vector delivers a codon-optimized *F9* gene with the FIX-Padua (R338L) variant under the control of a liver-specific LP1 promoter. Following hepatocyte transduction, endogenous FIX is produced. The recommended dose is as follows: 2 × 10^13^ gc/kg; re-dosing is currently not possible [34].

In the pivotal HOPE-B trial (NCT03569891), after a single infusion, the ABR for all bleeds decreased from 4.1 (lead-in on prophylaxis) to 1.9 during months 7–18 post-treatment (ABR ratio 0.46; 95% CI 0.26–0.81). Most patients discontinued prophylactic FIX. Mean FIX activity levels were ~37–42% of normal at months 6–24. Four-year updates confirm the durability of the effect in the majority of participants [34,35,36].

The most common adverse events were transient elevations of ALT/AST, managed with close monitoring and corticosteroids; infusion-related reactions were also reported. As with all AAV therapies, re-dosing is not possible; pre-existing or induced immune responses may reduce transgene expression. In patients at risk of HCC, regular liver surveillance is recommended [34].

Approved for adults with hemophilia B, fulfilling the label criteria requires careful post-infusion monitoring of liver function. Long-term follow-up is ongoing; challenges include inter-individual variability, lack of re-dosing options, and immune barriers [34].

In the pivotal HOPE-B trial (NCT03569891), a single dose of 2 × 10^13^ gc/kg was administered. The annualized bleeding rate (ABR) decreased from 4.1 to 1.9 (months 7–18 vs. lead-in), and most patients discontinued prophylaxis. The mean factor IX (FIX) activity remained at approximately 37–42% between months 6 and 24. The follow-up after more than four years confirmed the durability of the therapeutic effect in most patients [37,38,39,40].

#### 4.6.2. Beqvez (Fidanacogene Elaparvovec)

A single intravenous infusion of the AAVRh74var vector can carry the *F9* gene with the FIX-Padua (R338L) variant. The vector has strong hepatotropism, enabling long-term FIX expression. The recommended dose is as follows: 5 × 10^11^ vg/kg (approx. 60 min infusion). This requires negative testing for pre-existing neutralizing antibodies against the AAVRh74var capsid (FDA-approved companion test) [41].

In the phase 3 BENEGENE-2 trial (NCT03861273), treatment demonstrated superiority over prophylaxis: ABR 4.43 → 1.3 from week 12 to month 15 (−71%; *p* < 0.0001) and a −92% reduction in annual FIX infusions. Results published in NEJM confirmed a bleeding reduction and stable FIX expression [42,43,44].

Most common adverse events were transient transaminase elevations requiring monitoring and sometimes corticosteroids; infuse on-related reactions were also reported. Therapy requires the absence of anti-AAVRh74var antibodies; as with other AAV therapies, re-dosing is not possible. In February 2025, Pfizer discontinued global commercialization of Beqvez due to limited uptake [45].

The FDA approved Beqvez in April/May 2024 for adults with moderate-to-severe hemophilia B, fulfilling label criteria. Following Pfizer’s 2025 decision, market availability is limited, but long-term follow-up of participants (up to 15 years) continues [46,47].

In the pivotal BENEGENE-2 trial (NCT03861273), a single dose of 5 × 10^11^ vg/kg was administered. The annualized bleeding rate (ABR) decreased from 4.43 to 1.3 between week 12 and month 15, representing a 71% reduction, and FIX consumption was reduced by 92%. Superiority over prophylaxis was demonstrated, with stable FIX activity maintained throughout the observation period [42,47].

### 4.7. Immunological Challenges

Immune responses to the AAV capsid and neutralizing antibodies significantly limit the efficacy of gene therapy [10]. Studies have shown that even low antibody titers can prevent transgene expression. These immune responses can sometimes be suppressed with corticosteroids like prednisolone, but such treatment may not be safe for all patients [10].

### 4.8. Strategies to Overcome Immune Responses

Several approaches have been explored to overcome immune barriers in AAV-based gene therapy. One strategy involves the use of AAV capsids engineered with mutations—such as tyrosine-to-phenylalanine (Y-F) substitutions—that reduce capsid ubiquitination and subsequent degradation, thereby improving transgene expression and reducing immune recognition [48]. Another method is plasmapheresis, which physically removes circulating neutralizing antibodies from the bloodstream, potentially allowing for successful vector transduction [49]. Targeted delivery of the vector directly to an isolated segment of the liver via the portal vein has also been investigated, aiming to enhance hepatic uptake while limiting systemic exposure [12]. Pharmacologic immunosuppression, using agents like rituximab and cyclosporine A, may further mitigate adaptive immune responses [12]. Finally, the co-administration of empty AAV capsids as “decoys” can serve to saturate pre-existing antibodies, thereby preserving the therapeutic vector for effective gene delivery [12,50].

In conclusion, gene therapy for hemophilia B using AAV vectors has shown promising results, particularly with liver-directed delivery. However, immune challenges continue to limit the accessibility and effectiveness of this approach. Further research is needed to develop improved capsids, to optimize immunosuppression strategies, and to find solutions to overcome the barrier of pre-existing neutralizing antibodies [51].

### 4.9. Lentiviral and Retroviral Vectors

Lentiviral and retroviral vectors have emerged as promising alternatives for gene therapy in hemophilia B, primarily due to their ability to integrate into the host genome, enabling long-lasting transgene expression in proliferating cells—a feature not offered by episomal AAV vectors [12,52]. Initial use of γ-retroviruses was limited by their inability to transduce non-dividing cells and their potential for insertional oncogenesis, leading researchers to pivot toward lentiviral vectors (LVs) derived from HIV-1 [53]. LVs can efficiently infect both dividing and quiescent cells, and pseudotyping with envelope proteins—most commonly the VSV-G glycoprotein—allows adjustment of tissue targeting and biodistribution. In ex vivo strategies, hematopoietic stem cells (HSCs) are genetically modified using LVs carrying the F9 gene and then reinfused into the patient, resulting in stable FIX expression and immune tolerance [12]. Other approaches have targeted erythroid or platelet lineages with therapeutic outcomes, although they often require high-dose conditioning and pose immunogenicity concerns [54]. In vivo gene delivery to hepatocytes, the physiological site of FIX synthesis, has also proven feasible, though complications arise from non-specific tropism and transduction of antigen-presenting cells, which can elicit immune responses. To mitigate this, liver-specific promoters and regulatory elements, such as miR-142 target sequences, have been incorporated to confine expression and promote transgene-specific immune tolerance via regulatory T cells [55]. Notably, hepatic LV delivery can suppress pre-existing inhibitor responses to FIX. Still, innate immune reactions—including type I interferon signaling via TLR7 and the cGAS-STING pathway—continue to restrict transduction efficiency [56]. To reduce the risk of insertional mutagenesis, integrase-defective lentiviral vectors (IDLVs) have been developed. These vectors allow transient gene expression without permanent integration, making them safer, but they fall short of maintaining long-term expression in dividing tissues. Thus, while LVs and IDLVs show great therapeutic potential, they must overcome both immunological and genomic safety challenges before becoming a mainstream treatment for hemophilia B [12].

### 4.10. Other Non-Viral Gene Therapy Strategies

Non-viral gene therapy represents a promising alternative to viral vector-based approaches for treating hemophilia B, primarily by eliminating immune responses to viral proteins [12]. One of the simplest strategies involves the delivery of naked plasmid DNA, but its clinical utility is limited due to poor transfection efficiency and transient transgene expression. To address these limitations, researchers have explored transposon-based systems—particularly Sleeping Beauty (SB) and piggyBac—which enable stable genomic integration and prolonged expression of the F9 gene [57]. While animal studies have shown encouraging results, concerns remain about the risk of insertional mutagenesis due to random integration events. In small animal models, hydrodynamic injection is the most used delivery method for transposon systems, whereas larger animal studies have employed viral vectors, especially adenoviruses, to deliver transposons efficiently [12].

Advances in genome editing technologies—such as zinc-finger nucleases (ZFNs), transcription activator-like effector nucleases (TALENs), and CRISPR/Cas9—have facilitated more precise gene correction strategies, including targeted insertion into genomic “safe harbors” or direct repair of the defective F9 gene [58]. Dual-vector systems using AAVs to deliver both the therapeutic F9 gene and the editing nuclease (e.g., ZFN) have shown efficacy in correcting gene defects in both neonatal and adult mouse models. Despite these advancements, challenges remain related to limited vector packaging capacity and efficient gene delivery methods [12].

Innovative approaches, such as oral administration of plasmid F9 DNA encapsulated in chitosan nanoparticles, have also been tested. In hemophilia B mouse models, this strategy led to localized transgene expression in the small intestine and partial phenotypic correction of bleeding, although it failed to induce immune tolerance or eliminate pre-existing inhibitors [12]. In conclusion, non-viral gene therapy strategies for hemophilia B continue to evolve rapidly, combining novel delivery systems with precision genome editing to overcome the limitations of transient expression and immune reactivity [12].

Table 2 presents a comparison of gene therapy strategies in hemophilia (Table 2).

While most current reviews focus primarily on AAV-based vectors, a critical comparison across different gene therapy platforms reveals that each approach is associated with distinct immunological barriers. These challenges are particularly relevant in pediatric patients, where liver growth and immune maturity affect long-term efficacy, and in individuals with pre-existing immunity, who may be excluded from current trials. To address these aspects, we have constructed a comparative Table 3 summarizing the key immune barriers, strategies to overcome them, and special considerations across AAV, lentiviral, and CRISPR-based approaches (Table 3) [39,59,60,61,62,63,64].

Major clinical trials on gene therapy for hemophilia A (HA) and B (HB) are shown in Table 4.

## 5. Conclusions

Gene therapy for hemophilia has made remarkable progress over the past two decades, culminating in the approval of the first AAV-based treatments for both hemophilia A and B. Current evidence demonstrates that the durability of FIX expression in hemophilia B is superior and more consistent than FVIII expression in hemophilia A, which continues to decline over time. The field, therefore, faces three major hurdles: immune responses to AAV vectors, loss of FVIII expression, and the prohibitive cost and limited uptake of licensed therapies.

Future developments are likely to come from less immunogenic capsid designs, optimized FVIII constructs, and next-generation delivery platforms, such as CRISPR/Cas-based editing, lipid nanoparticles (LNPs), and ex vivo modified hepatocytes. These innovative approaches may overcome the durability gap and broaden the therapeutic landscape.

Importantly, future research must also focus on safely extending gene therapy to pediatric patients, who remain excluded from current protocols due to the risk of loss of efficacy in the developing liver. Another critical factor will be ensuring cost-effectiveness and equitable global access, without which the clinical impact of these breakthroughs will remain limited. Considering the pace of scientific and clinical progress, it is reasonable to expect that gene therapy may evolve into a standard of care for both hemophilia A and B within the next decade.

## Figures and Tables

**Table 1:** Gene therapy in hemophilia- comparison of benefits and risks [7,8,9].

Category	Benefits	Risks/Limitations
Efficacy	- Endogenous production of clotting factor - Significant reduction in bleeding episodes	- Variable efficacy among patients - Possible loss of effect over time - Currently, AAV-based gene therapies are not recommended for pediatric patients due to a theoretical loss of efficacy in the developing liver
Treatment mode	- Single intravenous administration	- Currently, no option for re-treatment with the same therapy (immune responses)
Quality of life	- Elimination or reduction in the need for regular infusions	- Need for long-term monitoring
Safety	- AAV vectors are relatively safe and typically do not integrate into the host genome	- Theoretical risk of cancer (DNA integration) - Possible immune reactions - The risk of genotoxicity/mutagenesis, particularly in relation to gene-editing approaches (e.g., CRISPR-based methods).
Costs	- Potential long-term savings (no ongoing therapy required)	- Very high one-time cost
Long-term effect	- Potential multi-year efficacy (even >8 years in some studies)	- Still lacking data from over 10 years of human follow-up

**Table 2:** Comparison of gene therapy strategies in hemophilia.

Strategy	Expression Efficiency	Oncogenic Risk	Immunogenicity	Duration of Effect	Re-Treatment Possible
AAV (liver-directed transfer)	High	Low	Moderate (capsid + transgene)	Years	No
Lentivirus (ex vivo)	High	Moderate–High	Low	Long-lasting	Yes
IDLV	Moderate	Low	Low	Short	Yes
Transposons (SB, piggyBac)	Moderate	Moderate	Low	Long-lasting	Yes
CRISPR/Cas9	High (if editing successful)	Low–High (method-dependent)	Low	Long-lasting	Not applicable
Plasmid DNA (naked DNA)	Low	None	Low	Short-term	Yes
LNP (lipid nanoparticles)	Low–Moderate	None	Low	Short	Yes

AAV—Adeno-Associated Virus; IDLV—Integrase-Defective Lentiviral Vector; SB—Sleeping Beauty (transposon system); piggyBac—piggyBac Transposon System; CRISPR/Cas9—Clustered Regularly Interspaced Short Palindromic Repeats/CRISPR-associated protein 9.

**Table 3:** Comparative overview of immunological barriers and strategies to overcome them across major gene therapy platforms (AAV, lentiviral, CRISPR), with special considerations for pediatric patients and individuals with pre-existing immunity [39,59,60,61,62,63,64].

Platform	Key Immunological Barriers	Strategies to Overcome
AAV (in vivo, liver-directed)	- Pre-existing neutralizing antibodies to capsid (20–40% prevalence) - CD8+ T cell responses causing hepatocyte clearance - Inability to re-dose	- Use of engineered capsids (Y-F mutations, novel serotypes) - Corticosteroids for liver enzyme elevations - Empty capsid decoys - Plasmapheresis or immunosuppression
Lentiviral (ex vivo HSC/in vivo approaches)	- Innate immune sensing via TLR7, cGAS-STING - Potential adaptive immune responses to transgene - Insertional mutagenesis triggering immune activation	- Ex vivo modification of autologous HSCs promotes immune tolerance - Use of tissue-specific promoters (liver, myeloid) - Development of integrase-defective vectors (IDLVs)
CRISPR/Gene Editing	- Innate immune recognition of Cas9 protein - Adaptive immunity against bacterial nucleases (Streptococcus pyogenes Cas9) - Inflammatory responses at off-target sites	- Development of humanized/smaller nucleases (SaCas9, Cas12a) - Transient delivery using mRNA or RNP complexes via LNPs - Careful off-target screening

**Table 4:** Major clinical trials on gene therapy for hemophilia A (HA) and B (HB) [45]. Accessed ClinicalTrials.gov on 31 Aug 2025 [65].

Hemophilia A			
Trial Details	Trial Details	Trial Details	Trial Details
Severe hemophilia A with pre-existing anti-AAV5 antibodies	NCT0352	BioMarin Pharmaceutical	Phase 1/2
Severe HA	NCT02576795	BioMarin Pharmaceutical	Phase 1/2
Valoctocogene roxaparvovec in hemophilia A	NCT03370913	BioMarin Pharmaceutical	Phase 3
Valoctocogene roxaparvovec + corticosteroids	NCT04323098	BioMarin Pharmaceutical	Phase 3
Evaluation of 4 × 10^13^ vg/kg valoctocogene roxaparvovec	NCT03391974	BioMarin Pharmaceutical	Phase 1/2
Gene therapy for HA	NCT03001830	University College London/MRC	Phase 1
AAV vector gene therapy: safety and dose escalation	NCT03370172	Baxalta	Phase 1/2
PF-07055480 in moderate/severe HA	NCT04370054	UniQure Biopharma BV	Phase 1/2
rAAV2/6-FVIII (SB-525) in severe HA	NCT03061201	Pfizer	Phase 1/2
Lentiviral FVIII gene therapy	NCT03217032	Shenzhen Geno-Immune Medical Institute	Phase 1
**Hemophilia B**			
AAV5-hFIXc in moderate/severe HB	NCT02396342	UniQure Biopharma BV	Phase 1/2
Dose confirmation of AAV5-hFIXco-Padua	NCT03489291	UniQure Biopharma BV	Phase 2b
HOPE-B: AMT-061 in moderate/severe HB	NCT03569891	UniQure Biopharma BV	Phase 3
Single-dose escalation of AAV8-FIX	NCT01687608	Baxalta	Phase 1/2
SHP648 (AAV vector) in HB	NCT04394286	Baxalta	Phase 1/2
Escalating-dose complementary AAV vector in HB	NCT00979238	St. Jude Children’s Research Hospital + collaborators	Phase 1/2
Long-term safety and efficacy of SPK-9001	NCT03307980	Pfizer	Phase 1/2
PF-06838435 in moderate/severe HB (BENEGENE-2)	NCT03861273	Pfizer	Phase 3
Lentiviral FIX gene therapy	NCT03961243	Shenzhen Geno-Immune Medical Institute	Phase 1

## Data Availability

No new data were created or analyzed in this study. Data sharing is not applicable to this article.
